# Unraveling Sorghum Allelopathy in Agriculture: Concepts and Implications

**DOI:** 10.3390/plants10091795

**Published:** 2021-08-28

**Authors:** M. Iftikhar Hussain, Subhan Danish, Adela M. Sánchez-Moreiras, Óscar Vicente, Khawar Jabran, Usman Khalid Chaudhry, Ferdinando Branca, Manuel J. Reigosa

**Affiliations:** 1 Department of Plant Biology and Soil Science, Universidad de Vigo, Campus Lagoas-Marcosende, 36310 Vigo, Spain; adela@uvigo.es (A.M.S.-M.); mreigosa@uvigo.es (M.J.R.); 2 CITACA, Agri-Food Research and Transfer Cluster, Campus da Auga, Universidad de Vigo, 32004 Ourense, Spain; 3 Department of Soil Science, Faculty of Agricultural Sciences and Technology, Bahauddin Zakariya University, Multan 60800, Pakistan; sd96850@gmail.com; 4 Institute for the Conservation and Improvement of Valencian Agrodiversity (COMAV), Universitat Politècnica de València, Camino de Vera s/n, 46022 Valencia, Spain; ovicente@upvnet.upv.es; 5 Department of Plant Production and Technologies, Faculty of Agricultural Sciences and Technologies, Niğde Ömer Halisdemir University, Niğde 51240, Turkey; khawarjabran@gmail.com; 6 Department of Agricultural Genetic Engineering, Faculty of Agricultural Sciences and Technologies, Niğde Ömer Halisdemir University, Niğde 51240, Turkey; ukojla0455@gmail.com; 7 Department of Agriculture, Food and Environment, Università degli Studi di Catania - Piazza Università, 95131 Catania, Italy; fbranca@unict.it

**Keywords:** weed suppression, allelochemicals, sorgoleone, benzoquinone, phenolics, cropping systems

## Abstract

Allelopathy is an ecological phenomenon that involves the production and release of biomolecules from different crops, cultivated plants, and bacteria or fungi into the soil rhizosphere and impacts other organisms in the vicinity. Sorghum possesses vital allelopathic characteristics due to which it produces and releases different biomolecules from its root hairs, stems, and grains. Several studies have reported that sorghum acts as an allelopathic crop, decreasing the growth and eco-physiological attributes of surrounding plants and weeds growing simultaneously or subsequently in the field. Sorghum allelopathy has been exploited in the context of green manure, crop rotations, cover crops, and intercropping or mulching, whereas plant aqueous extracts or powder might be an alternate method of weed control. A diverse group of allelochemicals, including benzoic acid, p-hydroxybenzoic acid, vanillic acid, ferulic acid, chlorogenic acid, m-coumaric acid, p-coumaric acid, gallic acid, caffeic acid, p-hydroxibenzaldehyde, dhurrin, sorgoleone, m-hydroxybenzoic acid and protocatechuic acid, have been isolated and identified from different plant tissues of sorghum and root exudates. These allelochemicals, especially sorgoleone, have been investigated in terms of their mode(s) of action, specific activity and selectivity, release in the rhizosphere and uptake and translocation in sensitive species. The present review describes the importance of sorghum allelopathy as an ecological tool in managing weeds, highlighting the most recent advances in the allelochemicals present in sorghum, their modes of action, and their fate in the ecosystem. Further research should focus on the evaluation and selection of sorghum cultivars with high allelopathic potential, so that sorghum allelopathy can be better utilized for weed control and yield enhancement.

## 1. Introduction

### 1.1. Weeds and Challenges to Modern Crop Production

The presence of weeds in agricultural fields decreases the quantity as well as the quality of the agricultural products, resulting in enormous financial losses for farmers [1]. Weeds are considered undesirable and detrimental plants that have harmful effects on the growth of desired plants and reduces the production potential of those desired plants. Weeds compete with crops for resources such as light, nutrients, space, and water, causing plant yields to suffer [2]. The presence of weeds is very common in crop plant fields, decreasing crop yields and increasing production costs, and consequently making crop production less cost-efficient [3]. Weeds cause a reduction of crop yields because of disturbances of plant growth due to allelopathy, competition, or both [4]. In recent decades, the use of herbicides to control weeds is causing severe problems and danger to the ecosystem, plants, and human beings. Meanwhile, long-term application of herbicides is the cause of generating resistance in weeds, which is currently becoming a serious problem in the development of sustainable agriculture worldwide. For example, triazines were one of the first popular groups of herbicides, which were applied widely due to their significant inhibition of the photosynthesis of various weeds [5,6,7]. The costs and unsustainability of current weed management are becoming increasingly apparent to farmers, to the public and to policymakers. This is evidenced by increasing demand for organic produce and recent discussions around banning widely used herbicides such as glyphosate [8]. Reduced reliance on chemical herbicides has led to searching for alternate natural products, such as diverse secondary metabolites, which could serve as lead compounds for weed management in the future [9]. The use of allelopathy may help improve plant and environmental productivity through the ecological management of weeds, pests, and plant diseases. In the last two decades, there has been a clear focus on plant-based natural products with the potential to replace chemical herbicides [10,11,12].

Allelopathy refers to the beneficial or harmful impact of one plant on its neighboring plants with the release of allelochemicals that influence their growth. Allelochemicals are a less toxic, safer, range of chemicals released by plants via volatilization, exudation, leaching, or residue decomposition [13]. Crop plants, such as soybean [14], sunflower [15], wheat [16], alfalfa [17], maize [18], sesame [19], rice [20], sorghum [21] and many others, have demonstrated allelopathic impacts on certain weed species. However, sorghum (*Sorghum bicolor* L.) is a well-known allelopathic crop that has the potential to suppress the weed growth due to the synthesis of sorgoleone [22,23]. It contains a range of allelochemicals, i.e., benzoic, p-hydroxybenzoic, vanillic, ferulic, chlorogenic, m-coumaric, p-coumaric, gallic, and caffeic acids [24], p-hydroxybenzaldehyde [25], dhurrin, sorgoleone [26], m-hydroxybenzoic acid and protocatechuic acid [27] with the potential to reduce weed growth. A comprehensive list of allelochemicals and secondary metabolites present in different plant parts of sorghum (roots, stems, foliage and panicle) is documented in Table 1. In sorghum, these allelochemicals are synthesized at greater concentrations in the adult stage of the plants [28]. Their toxicity can persist up to 22 to 28 weeks [29]. The allelochemicals are released into the soil rhizosphere during the plant life-cycle [30] or by the incorporation of crop debris, i.e., stubble [31] or stalk [32] into the soil. 

The mode of action of natural products includes multiple mechanisms, such as the reduction in percent and rate of germination together with reductions in root and shoot growth [33,34], interference with photosystem-II through electron transport [12], [35,36] and primary action on ATP production. In addition, this includes the inhibition of chloroplast oxygen evolution, a strong effect on mitochondrial function, alteration of nutrient absorption, chlorophyll pigments, carbon isotope discrimination [11,12], or water use efficiency [12,37]. The biochemical and physiological action mode of some common phenolic compounds on target plant species is shown in Table 2. 

### 1.2. Weed Management Strategies and Sorghum Allelopathy

Weeds are serious pests of plant species, and cause huge biological and economic crop losses, disrupt functioning, and suppress growth, development and yield of crops. The development of sustainable weed control strategies is urgently needed because of environmental pollution and evolution of herbicide resistance in weeds. Indeed, allelopathy is very important in natural, sustainable, and integrated weed management programs [58]. Sorgoleone, an allelopathic chemical secreted from Sorghum bicolor as root exudates in dryland, constitutes an excellent example of a natural herbicide [59]. At the juvenile stage, sorghum plants secrete significant concentrations of sorgoleone, reaching high concentrations in the root hairs (0.5 mg g−1 of root fresh weight) [22,60]. The potential of this allelopathic chemical is high in the suppression and inhibition of weed growth without disturbing the crop species [60]. It also offers an auspicious platform to spot its potential as a natural herbicide. Most broadleaf and grass weeds are susceptible to the herbicidal potential of sorgoleone. The persistence of sorgoleone is high in soil due to its hydrophobic nature and that it is absorbed by soil; thus, it possesses a long-term herbicidal activity effect that lasts for seven weeks after incorporation [61]. Sorgoleone directly affects the photosynthetic apparatus by disturbing the minerals and water uptake, especially in lower plants [62]. In addition to the above, it also inhibits electron transport in mitochondria and chloroplasts. The effectiveness of sorgoleone as an herbicide is comparable to synthetic herbicides for commercial use [63]. Allelochemicals released from sorghum plants have a direct influence on plant growth under laboratory, greenhouse and field experiments [10,64].

**Figure 1:**
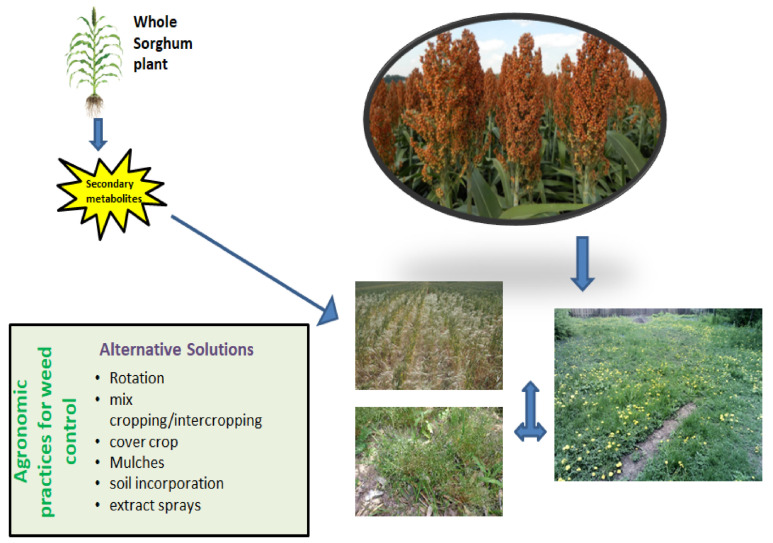
An illustration to demonstrate how sorghum can impact through several biological control practices on different weeds.

Allelochemicals secreted by sorghum plants directly influenced the growth of cultivated plants (such as rice and maize) in laboratory, greenhouse and field experiments [65,66]. Sorghum phytotoxicity differs with the plant organ, age, environmental factors, genotype and targeted weed species. Sorghum can be utilized in various ways to affect weeds, e.g., as surface mulch [67], by assimilation in soil [68], in aqueous extracts sprays [21], or by rotation [69], smothering [70] or mix cropping [71]. Figure 1 illustrates how sorghum can impact different weeds through several biological control practices. Suppressive effects on purple nutsedge density by incorporation of sorghum roots, stems and leaves in the soil have been reported by [72]. Similarly, foliar addition of a sorgaab (sorghum water extract) decreased the density and dry weight of purple nutsedge up to 44 and 67%, respectively, with an increase in maize grain yield of 44% [73]. Allelopathic effects of sorghum depend upon the genotype, age, location, environmental conditions and cropping system. 

The only study about the formulation of sorgoleone available is by Uddin et al. [74]. According to this study, it was wettable powder formulation with 4.6% active ingredient, i.e., sorgoleone; the formulation was prepared by blending methanol dissolved active ingredient with different carriers (e.g., kaolin 79.2%, SiO2 9.2%) and surfactant polyoxymethylene monooctadecyl ether. These authors reported that the germination process and seedling growth of *Setaria viridis* and *Aeschynomene indica* was decreased. Sorgoleone (0.2 g active ingredient (a.i.) L^−1^) completely reduced germination and seedling growth of broadleaf weeds (*Galium spurium, Rumex japonicus, Aeschynomene indica*, and *Amaranthus retroflexus*). A 20–25% inhibition was observed in weeds after application of sorgoleone as a post-emergence herbicide. Meanwhile, it was observed that sorgoleone 4.6 wettable powder (WP) is more effective in inhibiting the weed plant biomass and growth [74]. In an independent experiment, [64], the joint action of *Sorghum bicolor* (root exudate) and *Fagopyrum* spp. (root extract) on grasses (*Setaria viridis*) and broadleaf weeds (*Galium spurium, Rumex japonicus, Aeschynomene indica*, and *Amaranthus retroflexus*) under greenhouse conditions was observed A mixture of the two extracts (150 µg ml^−1^ of sorgoleone and 7.5 mg ml^−1^ of hairy root extract alone) significantly decreased germination and growth of target seedlings; among them, the broadleaf weeds, *Galium spurium, Aeschynomene indica, Rumex japonicus,* and *Amaranthus retroflexus* were the most susceptible. 

Allelopathic potential of root exudates of *Sorghum bicolor* on physiological traits of *Triticum aestivum* L., *Triticum durum* Desf., *Hordeum spontaneum* K. Koch., *Avena fatua* L. and *Phalaris minor* Retz. were studied [75]. They showed that *Phalaris minor* Desf. was the most sensitive in terms of reduced length, dry weight, and chlorophyll content as compared to untreated control. In another study, seedling growth of broadleaf weed species was suppressed more than grass weeds [76]. Weston et al. [77] published a comprehensive review on allelopathic potential and phytotoxicity of sorghum under laboratory, greenhouse and field conditions. They argued that allelochemicals in sorghum tissues vary depending on the plant parts, cultivars and age. The use of sorghum residues as green manure also induced adverse effects on weeds when incorporated as organic matter [78]. A variable class of polyphenols, such as dhurrin and sorgoleone, was also documented from sorghum roots, shoot and exudates [79]. 

The allelopathic potential of two sorghum varieties (Enkath and Rabeh) against different weeds was evaluated at 26.6 plant m^−2^ planting densities, assessing their effects on common purslane growth during 2009–2010 [80]. They found a significant reduction in weed root and shoot biomass (46–57%) compared to the control, following the treatment with sorghum. Sorghum cv. Enkath was more phytotoxic than cv. Rabeh. The main mechanism responsible for weed growth inhibition included extensive root growth of sorghum and allelochemicals released into the surrounding soil rhizosphere [80]. According to another study [81], sorghum accessions (353) from selected African countries (Botswana, Malawi, Mozambique, Namibia, Tanzania, South Africa, Zambia and Zimbabwe), showed significant variation of 334.62–584.69 μg mg^−1^ root fresh weight in production of sorgoleone. Among all the tested accessions, the South African landrace IS9456 produced the highest amount of sorgoleone (584.69 μg mg^−1^ root fresh weight), followed by an accession from Botswana and a wild sorghum accession from Zimbabwe. The authors concluded that wild sorghum varieties were superior in sorgoleone production compared to improved varieties and hence possess more phytotoxic potential against weeds [81]. The seeds were sown in pots, and sor1 gene expression was measured through RNA sampling from roots collected at 5, 10, 15, 20, 25, and 30 days after seedling emergence (DAE). In the inhibition test, cotton and three weeds were examined during single planting or planting with S. bicolor. The result showed an early expression of sor1 genes in several S. bicolor accessions by 5 days after emergence (DAE). Just one accession demonstrated the expression of sor1 up to 30 DAE. The plant biomass (roots and shoots dry weight) of spiny sandbur (*Cenchrus echinatus*) and Bermuda grass (Cynodon dactylon) was highly decreased. However, it is important to mention that the cotton intercropping with S. bicolor did not show any negative effects [82].

The allelopathic potential of sorghum has been demonstrated by several researchers in both laboratory and field studies [83,84], [21]. Three sorghum varieties (Hybrid sorghum IS41245 and GDLP 34-5-5-3) were evaluated to check their phytotoxicity and production of secondary metabolites such as sorgoleone [85]. Sorgoleone production and release of biological nitrification inhibition (BNI) activity by roots are strongly correlated (1 μg of sorgoleone is equivalent to 1 allylthiourea (ATU) activity). Soil nitrification was significantly inhibited by sorgoleone, and it was variety dependent. In this context, GDLP 34-5-5-3 and Hybrid sorgo exhibited greater production and release of sorgoleone and BNI than the variety IS41245 [85]. Sorgoleone is a hydrophobic molecule from the root hairs that exudates into the soil environment and affects the growth of weeds competing with sorghum [28]. The biosynthetic pathways of this molecule are relatively well known, except for some unknown enzymes. GC-MS analysis showed that the suppression of CYP71AM1 (P450 enzyme) in *S. bicolor* was mediated through RNAi and caused a decrease in sorgoleone production [86]. The authors concluded that CYP71AM1 contributes to the biosynthetic pathway of the allelochemical sorgoleone. Additionally, [87] also documented nitrification inhibition due to the release of allelochemicals from sorghum root hairs in the soil rhizosphere. The allelopathic potential of aqueous extract of two sorghum hybrids (Medovyi and Dovista) and the variety Sylosne 42, was evaluated against germination and seedling growth of *Beta vulgaris* L. and hybrid Ukrainian MS 97. Morphological traits, such as bud number, leaf length and plant height were highly reduced after 14 days of treatment. The results showed that the aqueous extract of Medovyi seeds was less phytotoxic than that of Sylosne 42 [88].

## 2. Role of Sorghum Allelopathy in Agro-Ecosystem 

The use of allelopathy in agricultural practices has been identified as a traditional means to control weeds and has become an important field of study [13]. One approach to utilize this development is to screen numerous crops and their cultivars for their allelopathic properties. Injurious after-effects of sorghum on subsequent crops have long been known to farmers without knowing the actual cause [89]. Experiments were conducted to evaluate the allelopathic effects of different crops, including sorghum [90]. They tested/screened various crops/plant species for their allelopathic effects. They found that sorghum was a highly allelopathic crop because its residues (allowed to decompose in the field) reduced the weed population up to 95%. Based on these studies, several scientific workers [91,92] proposed that crop residue of winter planted sorghum could be utilized for natural weed control.

Previously, Cheema [39] has worked on the allelopathic potential of sorghum in the field, and its possible use to control the weeds. He found that sorghum is a highly allelopathic crop, which exhibits effects on the subsequent crops in rotation, and it also influences weeds selectively. It was also observed that sorghum root residues, incorporated with soil, suppressed the growth (dry weight) of weeds such as *Chenopodium album*, *Phalaris minor*, *Avena fatua*, *Rumex dentatus*, *Senebiera didyma*, *Polygonum bellardi* and *Anagalis arvensis* by 20–48%, while purple nutsedge growth was decreased by 28 to 92%. On the contrary, the growth of *Melilotus parviflora* was promoted by the sorghum residues. It was noted that the amount of the material (sorghum) incorporated into the soil determined the observed effects, so that the greater the quantity, the stronger the allelopathic effect. 

Sorghum showed significant quantity of allelochemicals in stem, leaves and roots [38]. The chemical composition of sorghum residues showed significant concentration of phenolic acids, especially, p-coumaric acid, along with ferulic, syringic, vanillic and p-hydroxybenzoic acids. Subsequently, it was revealed that sorghum residues were significantly more toxic at the time of harvest and that it requires approximately 22–28 weeks to decompose [93]. Several phenolic compounds were identified from sorghum, including p-coumaric acid, m-hydroxybenzoic acid and protocatechuic acid as the principal inhibitors in sorghum roots [27], whereas dhurrin and sorgoleone were more important allelochemicals present in sorghum shoots [26]. Sorgoleone, which is released from the roots of living sorghum, is phytotoxic to several weeds, even at low concentrations [94]. Following these studies, Cheema [24] identified nine allelochemicals in sorghum herbage, namely benzoic, p-hydroxybenzoic, vanillic, m-coumaric, p-coumaric, gallic, caffeic, ferulic and chlorogenic acids, while some unknown compounds were also present in residues. Similarly, vanillic acid, p-hydroxybenzaldehyde, p-coumaric acid and ferulic acid were also detected in four sorghum hybrids, with p-coumaric acid present at a significantly higher concentration (7618 µg per g of plant dry weight) than ferulic acid [25]. 

Sorghum allelochemicals are produced either in the early seedling stage or near maturity. It was reviewed that phenolic acid concentration was higher at each growth stage [77]; even upon harvest a considerable amount of phenolic acid was observed [95]. The concentration of phenolic acid in young plants was again increased at the time of heading. Cheema [24] observed that whole-crop sorghum incorporated at the pre-flowering stage showed no allelopathic effects on wheat and weeds. However, the incorporation of mature sorghum roots, leaves and stems exhibited very strong allelopathic effects on the weeds and the wheat crop. In a later study, [95] found that the total phenol pool size of sorghum differed from 4 to 156 kg/ha in above-ground parts of the plant and from 1 to 16 kg/ha in roots. 

Allelopathic compounds of sorghum are species-specific and discriminatory in their action, i.e., they inhibit the growth of some species, but might not affect certain species and may have stimulatory effects on others [39]. The allelochemicals can also inhibit the sprouting and growth of seedlings [68]. Previous studies documented a primary action on ATP by sorgoleone, which then inhibits chloroplast and mitochondrial functions. Sorgoleone has the potential to block chloroplast function at the photosystem-II complex, whereas benzoic acid alters mineral uptake, chlorophyll content, photosynthesis, carbon flow and phytohormone activities. Inhibition of weeds through sorghum allelopathy resulted from the joint activity of various allelochemicals on various cell target sites was proposed by [40]. Gonzalez [41] also proved that sorgoleone is a strikingly intense inhibitor of electron transport in photosystem-II in both confined chloroplasts and PS-II layers. It is clear from the above information that sorghum allelochemicals affected most processes directly or indirectly related to growth. However, their effects were species-specific and concentration-dependent.

### Eco-Physiological Impact of Sorghum Allelochemicals

**Figure 2:**
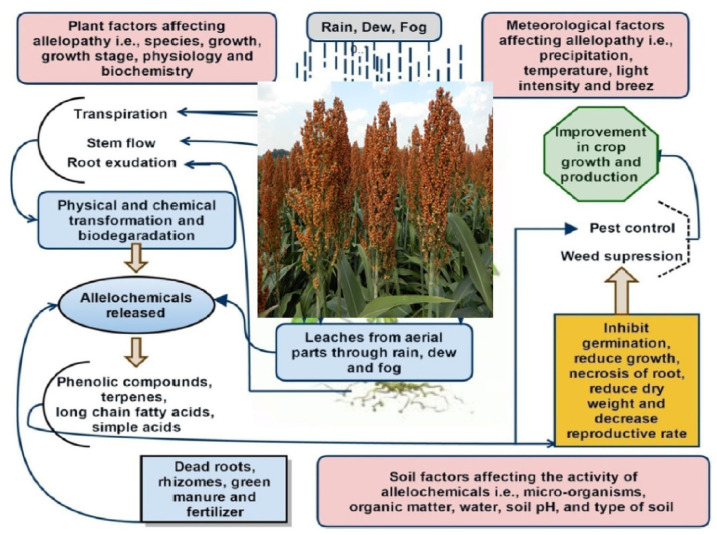
Overall processes of sorghum allelopathy in the soil environment and factors affecting allelopathy. The figure describes the way of allelochemical production from the plant parts, leachates from aerial parts, surface mulch, soil incorporation, spray of aqueous extracts, rotation, smothering, root exudates, and mix cropping.

Allelopathy phenomena include examples when one crop may destroy or encourage the germination, growth and yield of the associated crop(s) growing with it (crop mixtures or intercropping) or of the following crop (monoculture or crop rotations) through the release of leachates or washings from germinating seeds or decomposing crop residues [96]. Figure 2 shows an overall view of sorghum allelopathy, including the sources of allelochemicals production from plant parts, i.e., leachates from the aerial parts, surface mulch, soil incorporation, the spray of aqueous extracts, rotation, smothering, root exudates, or mixed cropping. Moreover, factors affecting allelopathy are also depicted. 

The germination and seedling growth of *Sphenostylis sternocarpa* (African Yam beans) was evaluated under the treatment of sorghum stem and maize roots aqueous extracts [97]. They reported that sorghum stem aqueous extracts had significant effect on radicle growth of both plants while degree of inhibition was increased with the increase in the concentrations of the extracts. Matos et al. [98] evaluated the bioherbicidal potential of sorghum carried out on *Cyperus rotundus* L. young seedlings with four types of sorghum extract: root extraction in alcohol, leaf extraction in alcohol, root extraction in water and leaf extraction in water, and five concentrations (0%, 20%, 40%, 80% and 100%). The results demonstrated that sorghum leaf extract had a significant impact on *C. rotundus* by interfering in plant growth attributes. The alcohol and aqueous extract showed significant growth retardation in *C. rotundus*, while leaf had more promising effects than roots [98]. 

Both extracts inhibited the tomato seed germination. In clay soil, *B. napus* extracts increased the bacterial population; however, *S. halepense* extracts restricted bacterial growth but stimulated fungal populations. Kim [99] explored the efficacy of allelochemicals from sorghum residues and water extracts and revealed that seed germination and the development of shoots and roots of crops such as radish, wheat, and rice were inhibited, while maize was less sensitive. The allelochemicals were extracted as fractions of chemical compounds such as methylene chloride, ethyl ether, hexane, and ethyl acetate. In another study, Ben-Hammouda [100] determined the variability of allelopathic effects among sorghum hybrids. Extracts obtained from different parts of the sorghum plant indicated considerable contrasts in phytotoxicity to wheat seedlings. Each extract exhibited a different level of phytotoxicity, which differed depending on the assayed hybrid and the performed measurement. The sorghum and sunflower water extracts (100% and 50% concentration), applied directly to wheat leaves at 30 days after sowing (DAS), increased the wheat grain yield by 5–14% over the control [101]. The maximum upturn in wheat yield (14%) was obtained in plots where 100 per cent sorghum water extract was sprayed, which was attributed to increased weed destruction and translocation of assimilates to the grain resulting from reduced competition. The extract treatments enhanced 1000-grain weight and the number of grains per spikelet, while the number of fertile tillers and spikelet length was reduced. The efficiency of sorgaab as a natural weed inhibitor was evaluated in Raya (Mustard) (*Brassica nigra*) [102]. They reported that the yield of the Raya crop was considerably increased (33–58%) over the control by applying one to three sprays of sorgaab. A significant effect of sorgaab treatment was also observed for plant height, the number of pods per plant and 1000-seed weight. Cheema and Khaliq [67] conducted experiments to explore the allelopathic effect of sorghum on mungbean. Applications of three sprays of sorghum water extract (at 15, 30 and 45 days after sowing (DAS)) and sorghum mulching at 10 and 15 t ha^−1^ increased the grain yield by 18.8, 7.2 and 12.8%, respectively, over the corresponding controls. This improvement was mainly attributed to a better weed control and enlarged leaf area, number of pods per plant and number of grains per pod. In another study, Cheema et al. [73] demonstrated that sorgaab foliar spray enhanced maize grain yields up to 13–44%, whereas the yield was increased by 36–40% when mature and chopped sorghum herbage was applied on the soil surface at the time of sowing. Likewise, three foliar sprays of sorgaab (sorghum water extract), applied after 15, 30 and 45 days of sowing were found to be effective in control of *Cyperus rotundus* L. in maize, as contrasted to hand weeding. Similarly, Cheema et al. [21] checked one and two foliar sprays of sorgaab against different varieties of wheat. The results showed that wheat grain yield was increased by 10–22%, and that leaf area, productive tillers, grain number, 1000-grain weight and harvest index were also improved. The cultivar Parwaz-94 was observed to be the most receptive to sorgaab, showing the largest increment in grain yield.

## 3. Sorghum Allelopathy and Sustainable Weed Management

Herbicide-resistant weeds are becoming increasingly competitive in agriculture systems, reducing the yield of most of the crops, particularly cereals and food grain crops [103,104,105,106,107]. Meanwhile, efforts are on the top agenda to include allelopathic crop cultivars, e.g., wheat, rice and sunflower that are yield stable and can also demonstrate phytotoxic influence on weeds [108]. Sorghum is an important grain crop with significant potential to suppress weeds under laboratory and field settings [84]. In less developed agriculture, weeds provide stiff competition to crops, thereby limiting crop growth, yield and economic profit [109]. 

Any inexpensive weed control measure would be helpful to farmers, hence, many plant species have been tested for their weed management potential, as they provide effective control by the suppression of the weed germination in agro-ecosystems. The effect of allelochemicals in *Sorghum bicolor* was previously reported [92]. Subsequently, numerous studies have been carried out to investigate the allelopathic potential of sorghum water extracts, sorghum mulch, and sorghum as cover crops on different weeds, and the reported results indicated that mature sorghum expressed selective, species-specific and concentration-dependent allelopathic effects [67,72,101,110]. The allelopathic impacts of sorghum on different weed species are documented in Table 3.

### 3.1. Use of Sorghum Water Extracts for Weed Suppression

Sorghum allelopathy has been used to control weeds in crop rotations [31] and intercropping systems [110] and by the use of sorghum mulches [32]. Similarly, the use of sorghum water extracts has shown significant suppression of weeds [67]. Allelopathic potential of water extracts were evaluated from different sorghum parts on weeds and crops in laboratory and greenhouse experiments [99]. They revealed that the allelopathic potential of sorghum was species-specific and relied upon source and concentration. Aqueous extract of sorghum leaves stems and roots significantly decreased the germination and seedling development of *Echinochloa colona* and radishes. They concluded that stem extract induced the most prominent inhibitory impact on *E. colona*, while each of the three extracts produced a similar reaction in radishes. In another study, [99] isolated toxic compounds of sorghum, and its chemical composition was resolved as far as their hindrance of germination and seedling development of *E. colona* and radishes. All hexane, ethyl ether, methylene chloride, ethyl acetic acid derivation and aqueous fractions were checked individually, and results showed that the ethyl ether fraction had the maximum inhibitory activity on *E. colona*. Of the eight fractions separated by rapid chromatography, the fraction with the dissolvable mixes of butanol: acetic acid: water (8:1:1) had the greatest lethality to plant species, *E. colona* and radish. Liquid chromatography coupled to mass spectrometry was used to identify the toxic compounds 1 methyl-1-(2-proponyl)-hydrazine, 1-aziridineethanol, 5-chloro-2-pentanone and 2-(methylseleno)-ethanamine. 

The feasibility of using aqueous extracts of allelopathic crops viz. sorghum and sunflower were investigated for weed control in wheat [101]. Spraying 100% water extracts of sorghum and sunflower after 30 days following wheat sowing diminished aggregate weed thickness up to 48% and 32% and whole weeds dry weight up to 51% and 51%, respectively. The weed biomass of *Rumex dentatus*, *Chenopodium album*, *Coronopus didymus*, and *Fumaria parviflora*, was reduced by 74%, 38%, 62% and 40%, respectively. Souza et al. (1999) evaluated the allelopathic impact of sorgoleone from sorghum root exudates upon *Phaseolus vulgaris* and *Amaranthus retroflexus*. Based on visual symptoms, *P. vulgaris* and *A. retroflexus* were the least and most susceptible species to sorgoleone, respectively. Root and shoot dry weights of *P. vulgaris* displayed an inversely proportional relationship with sorgoleone concentration. Khaliq et al. [114] sprayed sorgaab sorghum water extract that is obtained after soaking mature sorghum herbage in water for a period of one to two days for its weed control activity on soybean. Spraying of sorgaab at 25 and 45 DAS reduced the dry weight of all weeds by 20 to 42%, approximately, except that of *Trianthema portulacastrum*, which showed a yield increase of 9% over the control. Pendimethalin spray was also very effective in weed control but was more costly than sorgaab spray [114]. In another study, sorghum phytotoxicity was evaluated against various weeds in field-planted mungbean [121]. Plant dry biomass of target weeds (*Convolvulus arvensis* and *Portulaca oleracea*) decreased by about 60% and 75%, respectively, when treated with sorgaab foliar spray at 15, 30 and 45 DAS, while *Trianthema portulacastrum* remained unaffected [121]. Sorgaab reduced the weed thickness and dry weight by 32–62% and 47–75%, respectively, compared to the control, in raya crop [102]. Cheema et al. [121] conducted a field trial to observe the feasibility of sorghum allelopathy against the weed in traditional cotton. Sorgaab sprays decreased the total weed density by 13–54% and biomass by 87%. Cheema et al. [67] compared the concentration and frequency of sorgaab applications with hand weeding and chemical herbicide for controlling weeds in flooded wheat in a semi-arid district of Punjab. The dry weight and thickness of weeds were controlled by using sorgaab up to 35–49% and 22–46%, respectively, corresponding an increase in grain yield by 10–21%. Two foliar sprays of 10% sorgaab at 30 and 60 DAS were used to control the weeds in wheat with maximum yield. Chemical weedicides and the hand weeding technique were found to be wasteful for weed control because of higher costs in both cases.

Ahmad et al. [122] assessed the allelopathic potential of sorgaab as natural weed control in maize. Spraying of sorgaab suppressed the total weed density by 34–57% and horse purslane (*Trianthema portulacastrum*) density by 24–40%; the total dry weight reduction ranged from 13 to 34%, and that of horse purslane from 12 to 34%. In an independent study, Cheema et al. [21] used sorghum aqueous extracts as a foliar treatment against some winter weeds in four wheat varieties. One (30 DAS) and two (30 and 60 DAS) foliar applications of SWE impacted negatively the thickness and biomass of many weed species, such as *Chenopodium album*, *Phalaris minor*, *Avena fatua*, *Convolvulus arvensis*, and *Rumex dentatus*. On the other hand, the growth and density of *Melilotus parviflora* were improved. The obtained results showed that total biomass and weed thickness were significantly decreased. The Parwaz-94 variety was the most receptive to the aqueous sorghum extracts, showing the greatest increase in grain yield. The compound substances discharged by the plant deposits left on the dirt surface act uniquely in contrast to those released by the fused plant buildups.

### 3.2. Use of Sorghum Residues/Mulches for Weed Suppression

Weed growth could be suppressed by growing sorghum crops because the sorghum residues present on the soil surface release different allelochemicals which suppress the weed germination and seedling development [96]. The chemical substances released by the plant residues left on the soil surface respond differently than those released by plant residues incorporated into the soil. In the former case, they might be concentrated on the soil surface while, in the latter, the allelochemicals were diluted into the soil, following soil incorporation. Since the intensity of the allelopathic effect depends on the concentration of allelochemicals, their action is more intense on the soil surface under mulch [96]. On the other hand, when the release of these products is slower, the effects can be noticed for a more extended period. The higher the amount of plant material used for mulch, the greater is the total amount of allelochemicals present in the mulch and released, leading to a higher concentration of allelochemicals into the soil [123].

Allelopathic cover crops have been extensively used to inhibit weeds in organic agriculture. In this context, sorghum crop mulch and crop residues could contribute exceptionally to weed control [91]. Sorghum and sudan grass used as mulch resulted in reductions of weed biomass by approximately 90% and 85%, respectively. These authors concluded that the sorghum residues or mulches were allelopathic and could provide excellent suppression of several annual weeds. In another study, it was revealed that wheat, barley, oat, rye, sorghum and sudan grass mulch were very effective in the suppression of several weed species [92]. Seedling growth and biomass of purslane and smooth crab grass significantly decreased by 70% and 80%, respectively, following treatment with sorghum mulch. The residues of sorghum and sudan grass completely inhibited smooth grass seed development for 60 days, whereas wheat, oat, barley and rye residues likewise reduced the aggregate weed biomass up to 75%, and also the early season weed development. In a field trial, Cheema and Ahmad [72] demonstrated that the combination of whole sorghum plants or different sorghum parts, separated or blended, generally suppressed the growth of weeds, except for *Melilotus parviflora*, which was promoted. In situ integration of sorghum roots reduced the dry weight of other weeds by 26 – 49%. The sorghum’s allelopathic effects relied upon the phase of sorghum integration, the quantity of sorghum mass incorporated into the soil and its developmental stage. These experiments showed that sorghum residues could be adequately used to manage some of the weeds in wheat fields. 

The sorghum residues incorporation into the soil as surface mulch at 0, 0.5, 1.0, 2.0 and 3.0% w/v, showed that the efficacy of sorghum allelochemicals was species-specific and depended upon the source and concentration [99]. The sorghum stem residue considerably restricted the seed germination of *E. colona* and radishes, but not that of rice. The crop residues (maize, proso millet, safflower, grain sorghum and winter wheat) incorporation into soil inhibited the seedling growth in goat grass (*Aegilops triuncialis*), and to a lesser extent in winter wheat [124]. For instance, the residue of sorghum grain decreased seedling development of goat grass by 78% and that of winter wheat by 50%. The sorghum stem deposits impressively limited seed germination of *E. colona* and radishes, yet not that of rice. The chopped residues of four crops (sunflower, sorghum, rice and wheat) was incorporated in cotton fields at 5, 7.5 and 10 t ha^−1^ each [68]. Maximum reduction in weed population (ca. 52%) was observed in plots where wheat residue was applied at 5.0 t ha^−1^. This was followed by wheat (7.5 t ha^−1^), rice (7.5 t ha^−1^) and sorghum (10.0 t ha^−1^), with a reduction in the weed population with respect to the controls of about 40%, in all cases. Regarding dry weed biomass, the maximum reduction was observed in plots receiving sorghum crop residues at 10.0 t ha^−1^, amounting to 45.3% less than in the control.

Narwal et al. [70] observed the following order of weed suppression: pearl millet > maize > sorghum > cluster bean > cowpea. The residual suppression effect on weeds even persisted in the next crop. The sorghum herbage (applied as surface mulch at 10 and 15 t ha^−1^) in mungbean fields showed a significant reduction in the dry weight of *Trianthema partulacastrum* by 14 to 20% and 18 to 45%, respectively [32]; on the other hand, the reduction in thickness and dry weight of other weeds (*Cyperus rotundus*, *C. arvensis* and *P. oleracea*) was in the range of 52–68% and 60–77%, respectively. Cheema and Khaliq [67] studied the efficacy of allelochemicals of sorghum stalk integrated into the soil on rabi weeds and wheat crop. Mature sorghum chopped herbage (2 to 6 Mg ha^−1^) caused the reduction of weed dry weight by 20–41% and 42–56%, respectively, and an increase in wheat grain yield by 6 to 17%. In another study, [32] conducted a field trial to check the potential of sorghum allelochemicals to control the weeds in desi cotton, showing that sorghum mulching (3.5, 7.0, 10.5 t ha^−1^) suppressed the cumulative density of weeds by 23–62%, whereas 52–70% and 54–64% reductions were noted by using chemical treatment and hand weeding, respectively. The reduction in weed biomass under sorghum mulching was up to 56%.

### 3.3. Effect of Sorghum in Crop Rotation

Inclusion of sorghum in a rotation can help to control weeds through secretion of allelochemicals, which ultimately suppress the weeds. In a field trial in Nebraska, grain sorghum reduced the weed density, biomass and seedling growth in soybeans or maize [125]. In areas where sorghum has been included in the cropping system, weed infestation was constantly lower after few years with arrangements of four lines of grain sorghum with soybeans or maize [125]. Sorghum residues regularly delayed the growth of wheat crop; however, they did not influence yields, most likely due to the degradation of the allelochemicals in the soil over time [31]. No-till sorghum stover had little impact on stand establishment, yet every row decreased the yields of wheat grains, potentially on the grounds that allelochemicals drained gradually. In the rice-wheat crop rotation system, grain sorghum was cultivated before the rice planting. It was observed that this rotation with sorghum reduced the weed density in the succeeding rice crop with less herbicide application [69]. Likewise, the winter weeds may be controlled due to wheat replacement by oat and berseem clover (*Trifolium alexandrinum* L.).

### 3.4. Intercropping of Sorghum

Intercropping is a typical cultivation framework amongst livestock farmers of the emerging world. The main aim behind mixing harvests or planting in an adjacent grouping is to amplify, utilize and lessen the danger of crop disappointment. Intercropping maintains soil ripeness, reduces disintegration and may decrease insect harms. It has been guaranteed that one purpose behind this is the destruction of weeds [126]. Intercropping efficiency for weed control relies on the species consolidated, their relative extents and plant geometry in the field [127]. The output of intercropping frameworks can be decreased or improved, relying upon the inhibitory or stimulatory impacts of crops but ensuring that the other resources, such as light, nutrients, water and space are not limited [128]. In intercropping frameworks, the development and yield of segment crops increase because of more prominent supplement retention or better weed control than in harvests, yet the underlying mechanisms are not completely understood. Root exudates play a noteworthy part in the efficiency of crop mixtures as they may enhance crop development and yield of component crops through enhanced ion exchange, greater nutrient uptake and partial weed control, compared with pure crops [129,130].

#### 3.4.1. Allelochemicals Biosynthesis and Abiotic Stress Resistance

Plants as being sessile grow under natural environmental conditions where so many factors are involved for their nurturing. Therefore, any deviation from their required growth conditions at different growth stages exerts pressure [131]. Abiotic stresses are environmental adversities that negatively influence the plant growth and cellular functioning [132]. Abiotic stresses are the major hurdles in sustainable agriculture development. Currently, it is the main challenge for maintaining plant growth and crop productivity under such stress scenario for sustainable agriculture. All these environmental factors alone or in combination disrupt plant functions. Abiotic stresses are the chief cause of deprived yield and crop failure of sorghum [133]. Drought is one of the main abiotic stresses that is increasing at a rapid rate. A plant experiences drought once during its growth stage or throughout its life in certain regions. While living in the same biota, plants compete within their species and with species of other plant communities for nutrients and space. Allelochemicals are produced as tools for survival under these conditions. Survival of sorghum is difficult due to decline in available water resources, and there is a great need to adapt new strategies to grapple these stress factors. Plants produce phenolic acids in response to stress that work as osmoprotectants and antioxidants to scavenge oxidative stress [134]. Alteration in phenolic concentration is indispensable for plant survival. The exogenous application of phenolic acids helps plants in coping with harsh environmental conditions [135]. Moreover, phenolic acid is naturally a part of the allelochemicals that plants produce in high concentration with fluctuating environmental conditions [136]. Currently, studies are being carried out to observe the beneficial concentration of allelochemicals (phenolic acids) for the survival of plants and to protect them from environmental adversities. The residues of sorghum crop were used to extract water that resulted in inhibition of the germination and growth of the surrounding plants. This reduction in growth was due to phenolic acids, which are the characteristic feature of sorghum allelopathy [113]. Additionally, allelopathic sorghum was manipulated for the suppression of weed growth in wheat. The allelopathic plant extract (sorgaab) from sorghum was analyzed and it revealed higher concentrations of phenolic acids [137]. It has been reported that these phenolic compounds are among the plant secondary metabolites that are effective for abiotic stress tolerance in plants. Multifarious strategies can be adopted to cope with abiotic stresses, but sorgaab extraction from sorghum leaves proved to be efficient for minimizing the influence of adverse environmental factors [115].

#### 3.4.2. Production of Allelochemicals in Response to Abiotic Stresses

Allelochemicals have the potential to suppress the growth of weeds by disrupting water relations of plants in the root cell membrane. Additionally, they also result in biochemical changes for the alleviation of oxidative stress after their exposure to abiotic stresses [138]. Regardless of their benefits to cope with abiotic stresses, allelochemicals have not been given proper attention to explore their benefits to cope with environmental stresses [139]. Previous studies have explored potential groups of allelochemicals that confer stress tolerance. These studies assisted in bridging the gap of the positive role of allelochemicals that can exploited for stress resistance in sorghum [140]. However, the concentration of allelochemicals generally varies, as they are produced differentially during different growth stages, likewise the sensitivity of the plant against abiotic stresses also varies [141]. Allelochemicals that are produced in high concentrations in response to abiotic stresses include terpenoids and phenolic acids [142]. The synthesis of sorgoleone, dhurrin, and kinetin occurs in root, stem and leaves of sorghum and work as a first line of defense to alleviate abiotic stresses [143,144]. Higher accumulation of phenolic acids is positively correlated for abiotic stress tolerance of sorghum [137,145]. Allelochemicals are known to alleviate abiotic stresses. Numerous stress conditions alter the levels and synthesis of allelochemicals [146]. Fluctuations in temperature, decreased availability of water, and nutrient stress are the main environmental factors influencing the allelopathy. Additionally, herbicidal applications and heavy metals are also reported for differential regulation of allelochemicals [147]. Meanwhile, climatic factors also influence the synthesis of allelochemicals. It was reported that root growth of sorghum was influenced due to fluctuation in temperature, as optimum root growth goes along with sorgoleone production. The increase in temperature causes heat stress conditions that ultimately suppress the sorgoleone production in sorghum. The plants growing near the sorghum exert competition stresses that intensify the influence of abiotic stresses, resulting in decreased sorgoleone production [10]. 

#### 3.4.3. Stress Signaling by Allelochemicals in Sorghum

Abiotic stresses influence the transcriptional regulation of the allelochemicals. Sorghum growing under natural growth conditions is directly influenced by environmental stresses. Plants sense stress conditions and send signals to activate various molecular mechanisms in cells that resultantly cause physio-biochemical changes in plants to adapt to changed environmental conditions [148]. Plants even have the potential to send signals to neighboring plants with excessive production of allelochemicals under certain conditions [149]. Plants respond to stress signals by perceiving external harsh conditions and transmit between plant cells. The release of various type of allelochemicals such as soluble chemicals or volatile organic compounds helps in the regulation of soil microbes that confers a beneficial role by changing physio-chemical properties of the surroundings in the soil, which assist in inhibiting the growth of the competitor plants. As plants send signals to the neighboring plants, likewise, they also perceive beneficial signals from neighbors, which includes plant volatiles [150]. Abiotic stress signals in plants are perceived by increased levels of abscisic acid (ABA), calcium, and reactive oxygen species (ROS) that are commonly involved for some other pathways as well. Thereby, the response of allelopathic chemicals toward environmental pressure is likely to be related to elevated levels of ABA, calcium or ROS in plants [151]. 

Sorghum perceives environmental stresses and transmits a signal to the nucleus through complex cellular signaling networks that involves secondary messengers, i.e., calcium-associated proteins, reactive oxygen intermediates (ROIs), and mitogen-activated protein kinase (MAPK) cascades. The signaling network activates several transcriptional pathways that results in regulation of stress related genes resulting in physio-biochemical changes to protect the cellular membrane of plants [151,152].

Allelochemicals modify the mitogen-activated protein kinases (MAPK), which is the main enzyme for ethylene production [153]. The synthesis of allelochemicals occurs with the intervention of antioxidant enzymes that further assist in scavenging oxidative stress [154]. Moreover, these allelochemicals can trigger the gene expression pattern of root meristematic tissues that eventually assist root growth functions under stressful environments [83]. The literature shows great work has been done to understand the abiotic response of sorghum at molecular levels, but less work has been done to reveal the molecular basis of allelochemicals for conferring stress tolerance in sorghum.

#### 3.4.4. Genetic Factors Responsible for Sorgoleone Production

Natural products from plants offer a broad array of molecules with great diversity in their structure, biological activity and toxicologically, that can be used for managing weeds. The sorgoleone has been studied thoroughly [154,155,156]. Firstly, it was discovered during studying secondary metabolites that influenced the germination of witchweed [155]. It was noticed that allelochemicals can be absorbed by growing seedlings via hypocotyl and cotyledon, resulting in hindering the photosynthesis process. The sorgoleone sustain in soil for longer period than herbicides. Currently, studies are being done to identify the QTLs to enhance the production of sorgoleone in sorghum. Numerous studies have explored the biosynthetic pathway involved to produce sorgoleone [156]. Identification of genes controlling the production of allelochemicals would help in improving our knowledge regarding their synthesis pathways, release mechanisms into the soil rhizosphere, and corresponding phytotoxicity against different weeds. Genetic mechanisms responsible for the allelopathic effect of sorghum as a biological weed control are a new challenge, and fewer studies have focused on genetic factors. Recently, one of the studies by Shehzad et al. [157] highlighted that sorgoleone is not only a phenolic compound that contains allelochemical characteristics, but it also synthesizes other chemicals for the inhibition of the growth of neighboring plants. The SOR1 gene is responsible for sorgoleone production; it was reported that its higher transcript levels were observed from different root, stem and leaves of sorghum [43]. It was further confirmed from another study that showed that the higher expression of SOR1 resulted in weed suppression, and additionally the intercropping of sorghum and wheat exhibited no deleterious effect on cotton [82].

## 4. Conclusions and Future Perspectives

The study of the regulation of sorgoleone production by sorghum root hairs can increase the possibilities of employing sorghum as mulch or cover crop for effective management of germinating weed seedlings. The effect of sorgoleone resembles pre-emergent soil herbicides such as pendimethalin. Several researchers have proposed using a systematic approach employing candidate crops with better secondary metabolite profiles, and different agronomic techniques for better weed management under field settings. The phytotoxicity of sorghum and allelopathic interference has been elaborated under laboratory, greenhouse and in field trials. The present review also highlights the allelochemicals production under abiotic stresses, stress signaling by allelochemicals, and genetic factors responsible for sorgoleone production in sorghum. Different multidisciplinary approaches that incorporate sorghum crops for strategic weed control might be an alternative with great potential, using secondary metabolites that can also serve as lead compounds for herbicide discovery programs. These approaches should ideally have to be focused on weed control by employing agro-ecological and agronomic practices for better suppressing weeds at pre- and post-emergence stages, representing an alternative to genetically modified crops, which are considered by many (at least in the EU) as possibly harmful to the ecosystem and environment.

## Figures and Tables

**Table 1:** A comprehensive list of allelochemicals and secondary metabolites present in different parts (roots, stems, foliage and panicle) of *Sorghum bicolor* L.

Plant Species	Plant Parts	Secondary Metabolites	References
*Sorghum bicolor*	stems, leaves, roots	ferulic, p-coumaric, syringic, vanillic and p-hydroxybenzoic acids	[38]
*Sorghum bicolor*	roots	p-coumaric acid, m-hydroxybenzoic acid and protocatechuic acid	[27]
*Sorghum bicolor*	whole plant	benzoic acid, p-hydroxybenozoic acid, vanillic acid, m-coumaric acid, p-coumaric acid, gallic acid, caffeic acid, ferulic acid and chlorogenic acid	[39]
*Sorghum bicolor*	whole plant	vanillic acid, p-hydroxybenzaldehyde, p-coumaric acid and ferulic acid	[25]
*Sorghum bicolor*	roots	sorgoleone	[40,41]
*Sorghum bicolor*	stems	methyl-1-(2-proponyl)-hydrozine, 1-aziridineethanol, 5-chloro-2-pentanone and 2-(methylseleno)-ethanamine	[42]
			

**Table 2:** Biochemical and physiological mode of action of some of the common phenolic compounds on the target plant species, as reported in the literature.

Compounds	Mechanisms	Target Species	References
Ferulic and p-hydroxybenzoic acids	Inhibition of photosynthetic attributes	*Rumex acetosa*	[36]
Ferulic and p-hydroxybenzoic acids	Inhibition of relative water content, photosynthetic performance and carbon isotope discrimination	*Lolium perenne*	[12]
Ferulic, p-coumaric, o-hydroxyphenyl	Stimulation of chlorophyll degradation mechanism	*Oryza sativa*	[43]
acetic acid			
P-hydroxybenzoic acid	Inhibits seedling growth, induces water stress, stomatal closure		[44]
Hydroxyamic acid	Mitotic interference, inhibits seedling growth	*Lactuca sativa*	[45]
Caffeine	Inhibits cell division, abnormal root growth	*Zea mays*	[46]
Caffiec acid	Inhibits seed germination, plant growth, disruption of plant–water relationship, reduce chlorophyll contents	*Euporbia esula*	[47]
2-Benzoxazolinone (BOA)	Inhibits plasma membrane bound H^+^-ATPase in roots	*Avena fatua*	[48]
//	Inhibits germination, seedling growth, induces oxidative stress	*Lactuca sativa*	[49]
//	Disruption of plant–water relationship, adverse effect on transpiration and photosynthesis	*Lactuca sativa*	[45]
			[50]
Caffeic, p-coumaric, ferulic, salicylic acids	Induces water stress Glycine max,	*Sorghum bicolor*	[51]
Benzoic acid and cinnamic acid	Disruption of membrane or alter membrane permeability, efflux of ions, reduce chlorophyll content by damage of thylakoid membrane		[52]
Ferulic and p-hydroxybenzoic acids	Inhibition of photosynthesis, growth and carbon isotope discrimination	*Lactuca sativa*	[53]
Benzoxazolin-2(3*H*)-one (BOA) and cinnamic acid	Inhibition of leaf water content, photosystem-II efficiency, photon energy, photochemical quenching	*Dactylis glomerata, Lolium perenne, Rumex acetosa*	[35]
Cinnamic acid	Decrease of photochemical efficiency of PSII, quantum yield, fluorescence quenching, non-photochemical quenching, portion of absorbed photon energy thermally dissipated, photon energy absorbed by PSII antennae and trapped by “closed” PSII reaction centers, and carbon isotope composition	*Lactuca sativa*	[54]
Phenolic compounds	Reduction in hydraulic conductivity, net nutrient uptake	*Glycine max*	[52]
DIMBOA, MBOA	Inhibits seed germination	*Avena fatua*	[55]
p-coumaric, vanillic, ferulic acids	Inhibit photosynthesis and protein synthesis		[56]
Benzoxazolin-2(3*H*)-one, cinnamic acid	Reduction in leaf water relations, carbon isotopes discrimination, intrinsic water use efficiency	*Dactylis glomerata, Lolium perenne, Rumex acetosa*	[37]
p-hydroxybenzoic acid	Biochemical, physiological and isotopic traits inhibition	*Dactylis glomerata*	[57]

**Table 3:** Allelopathic effect of *Sorghum bicolor* L. alone and in association with other crops and their phytotoxic potential.

Plant Part	Phytotoxicity (% Reduction over Control)	References
(Sorghum Crop Residues)		
Sorghum root residues	25–50	[31]
Shoot extract	35–90	[100], [67,101] [111]
	
	
	
Shoot residues	42–98	[67]
	
	
	
	
Green manure, sorghum mulch	23–90	[31,92,112], [112]
	
	
	
	
Living roots	62–78	
Crop residue	29	[21]
Crop residue	35, 38, 49, 36	[32]
	23, 44	[113]
Sorghum	32, 35, 40	[32]
Sorghum	59	[114,115]
Joint action of Sorghum + other crop residues		
Sorghum herbage	23–41; 21–41	[116]
Sorghum +Eucalyptus	13–18; 28–32	//
Sorghum + Sunflower	30–35; 24–39	//
		//
Sorghum + Sesamum	21–24; 19–24	//
Sorghum + Tobacco	10–14; 14	//
Sorghum + Brassica	21–27; 28–24	//
Sorghum + Sunflower	36–55; 42–63	//
		//
Sorghum + Sunflower + Rice	18, 10, 17	[117]
Sorghum herbage	40	[118]
*Sorghum bicolor* × *Sorghum sudanese*		
Sorghum root residues	20–60	[29]
		[119]
Shoot extract	85–20	[120]
Shoot residues	25	[119] [92,112]
Green manure, sorghum mulch	0–30	[29,112]
Living roots	50–90	[119] [29]

## Data Availability

It was a review article. All the data has been mentioned in the manuscript.
